# Examination of Staphylococcus aureus Isolates from the Gloves and Gowns of Intensive Care Unit Health Care Workers

**DOI:** 10.1128/MRA.00691-20

**Published:** 2020-08-06

**Authors:** Timileyin Adedrian, Stephanie Hitchcock, Lyndsay M. O’Hara, Jane M. Michalski, J. Kristie Johnson, David P. Calfee, Loren G. Miller, Tracy H. Hazen, Anthony D. Harris, David A. Rasko

**Affiliations:** a Institute for Genome Sciences, University of Maryland School of Medicine, Baltimore, Maryland, USA; b Department of Microbiology and Immunology, University of Maryland School of Medicine, Baltimore, Maryland, USA; c Department of Pathology, University of Maryland School of Medicine, Baltimore, Maryland, USA; d Division of Infectious Diseases, Weill Cornell Medicine, New York, New York, USA; e LA Biomedical Research Center, Harbor-UCLA Medical Center, Torrance, California, USA; f Department of Epidemiology and Public Health, University of Maryland School of Medicine, Baltimore, Maryland, USA; Indiana University, Bloomington

## Abstract

Interactions with health care workers are often thought to be associated with the spread of microbes in the hospital setting. We have examined the genomic diversity of methicillin-resistant Staphylococcus aureus isolates from the gloves and gowns of health care workers from four hospitals in three states.

## ANNOUNCEMENT

This study examines 95 methicillin-resistant Staphylococcus aureus (MRSA) isolates obtained from the gloves and gowns of health care workers in the intensive care units of four hospitals in three states, Maryland, California, and New York, between January 2016 and August 2018. These isolates were collected as part of a study to examine the requirements for transmission of S. aureus isolates from colonized or infected patients to the gloves and gowns of health care workers during examined patient contacts (1). Each isolate has a matching patient isolate that was described previously (2). Samples were collected under University of Maryland School of Medicine Institutional Review Board approval number HP-00066759. Each swab sample was enriched overnight in tryptic soy broth with 6.5% salt (Becton, Dickinson, Sparks, MD) and plated on CHROMagar Staph aureus medium (Becton, Dickinson, Sparks, MD). All rose/mauve colonies were confirmed as S. aureus by Staphaurex latex agglutination and were confirmed as MRSA by susceptibility testing following the Clinical and Laboratory Standards Institute guidelines (3). A total of 95 Staphylococcus aureus isolates were collected and examined by whole-genome sequencing.

Genomic DNA was isolated from cultures that had been grown overnight in lysogeny broth. DNA was extracted in a 96-well format from 100 μl of sample using the MagAttract PowerMicrobiome DNA/RNA kit (Qiagen, Hilden, Germany) automated on a Hamilton Microlab STAR robotic platform. Bead disruption was conducted on a TissueLyser II instrument (20 Hz for 20 min) in a 96-deep-well plate in the presence of 200 μl phenol-chloroform. Genomic DNA was eluted in 90 μl water after magnetic bead cleanup. The resulting genomic DNA was quantified with PicoGreen. The sequencing libraries were generated with the KAPA HyperPrep kit (catalog number KK8504) and sequenced on the Illumina NovaSeq platform using a 2 ×150-bp paired-end read kit.

The total number of reads generated for each isolate is listed in Table 1; values averaged 2,855,320 bp per genome. All software was used with default values. Raw sequencing reads were filtered to remove contaminating phiX reads using BBDuk of the BBTools software suite (https://sourceforge.net/projects/bbmap). The raw reads were also filtered to remove contaminating Illumina adaptor sequences and quality trimmed using Trimmomatic v.0.36 (4). The resulting filtered reads were assembled using SPAdes v.3.13.0 (5). The assemblies were then filtered to contain only contigs longer than 500 bp with a k-mer coverage of ≥5×. Genomes containing more than 500 contigs or an aberrant GC content were removed from further analysis.

**TABLE 1:** Genome characteristics

Isolate	No. of reads	No. of bases sequenced	Genome coverage (×)	No. of contigs	Genome size (bp)	GC content (%)	*N*_50_ (bp)	GenBank accession no.	SRA accession no.
MRSA2_6GL	4,524,590	683,213,090	240	49	2,852,532	32.73	151,298	JABMEH000000000	SRR11787465
MRSA6_1GL	4,146,886	626,179,786	218	34	2,870,003	32.61	284,166	JABMEI000000000	SRR11787464
MRSA1_1GL	4,233,804	639,304,404	229	30	2,791,324	32.62	375,055	JABMEJ000000000	SRR11787453
MRSA8_2GL	4,358,228	658,092,428	225	38	2,930,504	32.68	296,619	JABMEK000000000	SRR11787412
MRSA15_6GL	3,521,866	531,801,766	187	38	2,841,414	32.75	214,284	JABMEL000000000	SRR11787385
MRSA18_3GOWN	4,587,088	692,650,288	245	43	2,826,119	32.62	299,642	JABMEM000000000	SRR11787374
MRSA22_1GL	3,084,810	465,806,310	162	47	2,868,533	32.75	221,190	JABMEN000000000	SRR11787427
MRSA25_6GL	3,643,810	550,215,310	194	48	2,843,327	32.72	316,045	JABMEO000000000	SRR11787400
MRSA27_2GL	5,527,524	834,656,124	304	29	2,742,918	32.73	222,216	JABMEP000000000	SRR11787389
MRSA31_1GOWN	3,759,116	567,626,516	196	34	2,897,784	32.66	304,504	JABMEQ000000000	SRR11787440
MRSA33_10GL	4,016,646	606,513,546	216	31	2,810,080	32.61	299,349	JABMER000000000	SRR11787463
MRSA40_1GOWN	4,551,104	687,216,704	237	53	2,894,939	32.74	419,332	JABMES000000000	SRR11787462
MRSA50_1GOWN	4,288,060	647,497,060	222	88	2,914,909	32.7	74,035	JABMET000000000	SRR11787461
MRSA53_4GL	3,636,304	549,081,904	191	44	2,867,763	32.77	419,331	JABMEU000000000	SRR11787460
MRSA55_1GL	2,873,984	433,971,584	147	29	2,955,377	32.6	381,526	JABMEV000000000	SRR11787459
MRSA59_5GL	3,531,496	533,255,896	187	36	2,857,580	32.71	243,700	JABMEW000000000	SRR11787458
MRSA61_2GOWN	4,173,858	630,252,558	216	28	2,913,513	32.64	219,529	JABMEX000000000	SRR11787457
MRSA62_6GOWN	4,225,696	638,080,096	222	31	2,869,261	32.61	304,667	JABMEY000000000	SRR11787456
MRSA66_6GL	4,675,570	706,011,070	250	33	2,827,657	32.61	284,067	JABMEZ000000000	SRR11787455
MRSA69_2GL	4,691,304	708,386,904	249	33	2,843,336	32.75	222,066	JABMFA000000000	SRR11787454
MRSA70_10GL	4,066,362	614,020,662	224	30	2,739,032	32.69	205,013	JABMFB000000000	SRR11787452
MRSA75_2GL	3,282,984	495,730,584	172	38	2,888,017	32.77	221,197	JABMFC000000000	SRR11787451
MRSA76_8GL	4,688,130	707,907,630	247	32	2,867,186	32.61	345,465	JABMFD000000000	SRR11787450
MRSA78_1GL	4,132,544	624,014,144	220	25	2,835,618	32.65	374,307	JABMFE000000000	SRR11787449
MRSA79_1GL	3,914,286	591,057,186	203	36	2,911,809	32.64	284,056	JABMFF000000000	SRR11787448
MRSA83_2GL	1,182,604	178,573,204	62	26	2,873,181	32.61	382,036	JABMFG000000000	SRR11787417
MRSA89_3GOWN	3,447,888	520,631,088	182	36	2,867,517	32.62	209,590	JABMFH000000000	SRR11787416
MRSA90_1GL	5,019,222	757,902,522	269	38	2,816,987	32.7	387,589	JABMFI000000000	SRR11787415
MRSA96_10GL	4,039,434	609,954,534	211	27	2,886,863	32.66	405,203	JABMFJ000000000	SRR11787414
MRSA104_1GL	3,044,344	459,695,944	160	31	2,868,336	32.61	345,396	JABMFK000000000	SRR11787413
MRSA106_10GL	4,073,250	615,060,750	222	34	2,770,570	32.67	222,208	JABMFL000000000	SRR11787411
MRSA110_4GL	3,706,078	559,617,778	196	40	2,850,295	32.71	215,893	JABMFM000000000	SRR11787410
MRSA123_1GL	4,219,330	637,118,830	230	33	2,765,385	32.68	236,734	JABMFN000000000	SRR11787409
MRSA134_10GL	4,312,818	651,235,518	226	34	2,881,801	32.66	209,899	JABMFO000000000	SRR11787408
MRSA136_1GL	5,131,588	774,869,788	265	28	2,918,655	32.64	381,526	JABMFP000000000	SRR11787407
MRSA135_10GL	4,247,700	641,402,700	221	32	2,907,741	32.64	304,501	JABMFQ000000000	SRR11787406
MRSA137_10GL	3,925,658	592,774,358	209	33	2,838,582	32.73	293,060	JABMFR000000000	SRR11787405
MRSA145_1GL	3,901,596	589,140,996	202	45	2,911,373	32.75	221,191	JABMFS000000000	SRR11787404
MRSA146_10GL	4,443,912	671,030,712	244	25	2,747,586	32.74	299,928	JABMFT000000000	SRR11787403
MRSA150_6Gown	3,203,830	483,778,330	169	36	2,855,927	32.6	223,549	JABMFU000000000	SRR11787402
MRSA152_10GL	3,785,196	571,564,596	201	36	2,844,245	32.74	428,845	JABMFV000000000	SRR11787384
MRSA159_4GOWN	6,006,876	907,038,276	316	26	2,866,938	32.61	345,457	JABMFW000000000	SRR11787383
MRSA161_5GL	4,626,044	698,532,644	246	36	2,837,103	32.76	469,521	JABMFX000000000	SRR11787382
MRSA163_7GOWN	3,658,216	552,390,616	196	32	2,819,135	32.77	243,944	JABMFY000000000	SRR11787381
MRSA167_9GL	4,057,940	612,748,940	218	27	2,808,181	32.7	243,973	JABMFZ000000000	SRR11787380
MRSA169_2GL	4,638,068	700,348,268	247	33	2,831,114	32.74	419,318	JABMGA000000000	SRR11787379
MRSA170_2GL	3,973,634	600,018,734	210	63	2,856,864	32.62	143,025	JABMGB000000000	SRR11787378
MRSA171_1GL	4,237,196	639,816,596	227	36	2,813,987	32.68	222,055	JABMGC000000000	SRR11787377
MRSA177_9GOWN	4,418,818	667,241,518	233	25	2,864,757	32.61	345,438	JABMGD000000000	SRR11787376
MRSA180_1GL	3,833,598	578,873,298	201	25	2,878,157	32.65	305,137	JABMGE000000000	SRR11787375
MRSA188_6GL	6,162,624	930,556,224	330	30	2,817,408	32.77	419,310	JABMGF000000000	SRR11787373
MRSA194_4GOWN	3,688,360	556,942,360	198	28	2,812,508	32.61	295,184	JABMGG000000000	SRR11787372
MRSA197_1GL	4,844,752	731,557,552	257	36	2,844,024	32.75	221,191	JABMGH000000000	SRR11787371
MRSA202_4GL	4,011,992	605,810,792	210	38	2,881,199	32.66	305,136	JABMGI000000000	SRR11787370
MRSA206_3GOWN	3,539,144	534,410,744	188	41	2,837,873	32.64	287,014	JABMGJ000000000	SRR11787433
MRSA228_2GL	3,712,452	560,580,252	192	29	2,919,777	32.63	381,525	JABMGK000000000	SRR11787432
MRSA237_1GL	4,194,822	633,418,122	221	29	2,864,946	32.6	333,129	JABMGL000000000	SRR11787431
MRSA243_4GL	3,797,642	573,443,942	208	27	2,753,113	32.69	226,146	JABMGM000000000	SRR11787430
MRSA244_1GOWN	3,831,102	578,496,402	202	31	2,867,119	32.61	304,503	JABMGN000000000	SRR11787429
MRSA250_1GL	4,588,244	692,824,844	242	27	2,868,270	32.62	308,333	JABMGO000000000	SRR11787426
MRSA252_3GL	3,425,350	517,227,850	180	39	2,865,654	32.76	221,191	JABMGP000000000	SRR11787425
MRSA260_10GL	3,693,330	557,692,830	192	26	2,910,300	32.63	379,551	JABMGQ000000000	SRR11787424
MRSA702_1GL	3,311,932	500,101,732	172	53	2,899,256	32.7	289,414	JABMGR000000000	SRR11787423
MRSA703_10GL	3,548,196	535,777,596	197	32	2,721,591	32.69	200,887	JABMGS000000000	SRR11787422
MRSA705_1GL	4,523,290	683,016,790	237	28	2,881,127	32.7	287,929	JABMGT000000000	SRR11787421
MRSA255_2GOWN	4,782,256	722,120,656	252	32	2,860,693	32.62	264,712	JABMGU000000000	SRR11787420
MRSA504_7GL	4,646,030	701,550,530	246	28	2,849,435	32.62	381,190	JABMGV000000000	SRR11787419
MRSA265_2GOWN	3,030,896	457,665,296	164	32	2,783,852	32.75	213,774	JABMGW000000000	SRR11787418
MRSA708_1GL	5,152,506	778,028,406	274	38	2,837,275	32.72	220,191	JABMGX000000000	SRR11787401
MRSA713_8GL	4,532,424	684,396,024	232	66	2,948,225	32.71	289,646	JABMGY000000000	SRR11787399
MRSA268_8GL	2,747,628	414,891,828	145	32	2,868,608	32.61	381,205	JABMGZ000000000	SRR11787398
MRSA508_10GOWN	4,334,190	654,462,690	230	56	2,846,577	32.66	180,937	JABMHA000000000	SRR11787397
MRSA509_1GOWN	4,795,002	724,045,302	249	71	2,906,582	32.62	94,952	JABMHB000000000	SRR11787396
MRSA720_10GL	4,491,500	678,216,500	234	29	2,895,673	32.68	442,377	JABMHC000000000	SRR11787395
MRSA274_2GOWN	4,259,436	643,174,836	227	35	2,834,588	32.76	469,494	JABMHD000000000	SRR11787394
MRSA277_10GL	4,687,412	707,799,212	246	28	2,871,449	32.6	305,050	JABMHE000000000	SRR11787393
MRSA281_2Gown	4,286,508	647,262,708	225	30	2,878,877	32.64	333,124	JABMHF000000000	SRR11787392
MRSA282_3GL	4,273,290	645,266,790	222	34	2,910,537	32.63	381,982	JABMHG000000000	SRR11787391
MRSA286_8GL	5,170,622	780,763,922	273	42	2,863,053	32.6	333,116	JABMHH000000000	SRR11787390
MRSA733_3GL	4,300,494	649,374,594	222	32	2,922,418	32.64	210,561	JABMHI000000000	SRR11787388
MRSA290_9GL	5,094,942	769,336,242	263	28	2,920,937	32.63	333,124	JABMHJ000000000	SRR11787387
MRSA292_5GL	4,386,620	662,379,620	228	36	2,906,872	32.62	381,159	JABMHK000000000	SRR11787386
MRSA520_2GL	4,131,570	623,867,070	221	47	2,817,654	32.62	190,099	JABMHL000000000	SRR11787447
MRSA739_1GOWN	4,921,200	743,101,200	258	63	2,879,352	32.71	387,685	JABMHM000000000	SRR11787446
MRSA294_10GL	4,017,506	606,643,406	218	23	2,782,834	32.62	374,288	JABMHN000000000	SRR11787445
MRSA297_6GL	5,124,906	773,860,806	277	35	2,788,735	32.66	267,802	JABMHO000000000	SRR11787444
MRSA300_1GL	4,578,492	691,352,292	241	44	2,867,697	32.79	215,700	JABMHP000000000	SRR11787443
MRSA524_1GOWN	3,899,822	588,873,122	209	57	2,817,788	32.63	172,216	JABMHQ000000000	SRR11787442
MRSA527_7GOWN	4,775,202	721,055,502	266	35	2,707,933	32.68	148,991	JABMHR000000000	SRR11787441
MRSA744_1GL	3,224,140	486,845,140	168	29	2,906,005	32.64	347,420	JABMHS000000000	SRR11787439
MRSA745_6GL	3,687,668	556,837,868	192	34	2,894,415	32.68	289,415	JABMHT000000000	SRR11787438
MRSA535_7GOWN	3,407,236	514,492,636	177	30	2,914,051	32.64	223,446	JABMHU000000000	SRR11787437
MRSA536_1GL	3,474,314	524,621,414	182	40	2,879,355	32.74	443,376	JABMHV000000000	SRR11787436
MRSA750_2GL	2,991,212	451,673,012	159	38	2,836,261	32.72	279,245	JABMHW000000000	SRR11787435
MRSA541_5GL	3,262,716	492,670,116	172	28	2,871,707	32.6	382,034	JABMHX000000000	SRR11787434

Relevant statistics, including GenBank accession numbers for assemblies and SRA links for each genome assembly, are included in Table 1. The genomes have a mean sequencing coverage of 198× (standard deviation, 58×; minimum, 58×; maximum, 1,008×). The final assemblies have a mean contig count of 36.2 contigs (standard deviation, 11 contigs; minimum, 23 contigs; maximum, 88 contigs), a mean genome size of 2,855,320 bp (standard deviation, 49,999 bp; minimum, 2,707,933 bp; maximum, 2,955,377 bp), a mean GC content of 32.7% (standard deviation, 0.06%; minimum, 32.6%; maximum, 32.79%), and a mean *N*_50_ value of 293,541 bp (standard deviation, 84,608 bp; minimum, 74,035 bp; maximum, 469,521 bp).

Further functional analysis will assess whether the S. aureus isolates contain genetic determinants that may promote transmission from patients to health care worker gloves or gown and possible transmission to subsequent patients.

### Data availability.

All data have been released, and accession numbers are listed in Table 1.
